# Combined intermittent theta-burst stimulation and functional electrical stimulation as adjuncts to intrathecal baclofen in hereditary spastic paraplegia type 11: a case report

**DOI:** 10.3389/fresc.2026.1942850

**Published:** 2026-10-08

**Authors:** Haodong Zhang, Jie Shen, Yanping Qu

**Affiliations:** Department of Rehabilitation Medicine, Shanghai Eighth People's Hospital, Shanghai, China

**Keywords:** case report, functional electrical stimulation, hereditary spastic paraplegia, intermittent theta-burst stimulation, intrathecal baclofen

## Abstract

**Background:**

Spastic paraplegia type 11 (SPG11) is the most common autosomal recessive subtype of hereditary spastic paraplegia, and is characterized by progressive corticospinal tract degeneration, lower-limb spasticity, and gait impairment. Intrathecal baclofen (ITB) effectively alleviates spasticity, but fails to reverse upstream neural pathway damage, resulting in a “functional ceiling” in motor recovery. Intermittent theta-burst stimulation (iTBS) and functional electrical stimulation (FES) have shown potential for enhancing neuroplasticity; however, their combined use as adjuncts to ITB in SPG11 remains unreported.

**Case presentation:**

A 23-year-old female patient with genetically confirmed SPG11 was admitted 2 weeks after ITB pump implantation with severe lower-limb spasticity, an inability to walk independently, and mild anxiety and depression. The patient underwent a 3-month combined intervention comprising iTBS, FES, and conventional rehabilitation, with dynamic interdisciplinary titration of the ITB dosage. Outcomes were assessed using the Modified Ashworth Scale (MAS), Manual Muscle Testing (MMT), the Berg Balance Scale, center of pressure analysis, the Hamilton Anxiety Scale (HAMA), and the Hamilton Depression Scale (HAMD).

**Outcomes:**

After 3 months of intervention, the optimal ITB dose was determined to be 62.82 μg/d. The MAS scores of both legs progressively declined, accompanied by increased MMT grades and improved standing balance. The HAMA and HAMD scores were markedly reduced. No adverse events occurred during the treatment.

**Conclusions:**

The combination of iTBS and FES as adjuncts to ITB was associated with improvements in motor function, balance, and psychological status in patients with SPG11. Dynamic interdisciplinary titration of the ITB dose may be important for balancing spasticity control and motor learning.

## 1 Introduction

Hereditary spastic paraplegia (HSP) is a heterogeneous group of neurodegenerative disorders defined by progressive length-dependent degeneration of the corticospinal tracts (CST), with a global prevalence of approximately 0.1 to 9.6 per 100,000 individuals and a gradually evolving clinical course (1). Based on inheritance patterns, HSP is categorized into autosomal dominant, autosomal recessive, and X-linked forms (2). Under the Harding clinical classification, HSP is divided into “pure” subtypes, characterized by spasticity and weakness confined to the lower limbs, and “complex” subtypes, which present with additional motor features (e.g., ataxia, dysarthria, peripheral neuropathy) and non-motor features (e.g., cognitive impairment, dorsal column degeneration, seizures, urinary symptoms) (3). SPG11 is the most frequently implicated gene in autosomal recessive HSP, accounting for approximately 20% of all recessive cases (4, 5). These progressive multisystem deficits severely impair activities of daily living. To date, no disease-modifying therapy is available; consequently, clinical care remains predominantly symptomatic (6).

Spasticity, a positive feature of the upper motor neuron syndrome, is a primary disabling symptom and a core therapeutic target in the management of HSP (7). Oral baclofen, a *γ*-aminobutyric acid type B receptor agonist, is the first-line pharmacotherapy for spasticity, but its clinical efficacy is limited by poor blood-brain barrier penetration. High oral doses are required to achieve therapeutic concentrations in the central nervous system, which frequently cause systemic adverse effects such as drowsiness, dizziness, and generalized weakness (8). Conversely, intrathecal baclofen (ITB) therapy involves the surgical implantation of a pump for the direct delivery of baclofen into the spinal subarachnoid space, bypassing the blood-brain barrier to achieve high spinal drug concentrations with minimal systemic exposure (9). Previous studies have confirmed that ITB significantly reduces spasticity and improves mobility in patients with HSP, making it a standard treatment for severe refractory spasticity (10, 11). However, ITB only targets spinal-level spasticity and cannot reverse CST degeneration or cortical dysfunction, resulting in a “functional ceiling” in which spasticity reduction translates into limited gains in independent mobility.

Recent studies have demonstrated that noninvasive brain stimulation (NIBS) techniques, such as transcranial magnetic stimulation (TMS), can enhance neural plasticity and improve motor function (12, 13). Increasing evidence indicates that NIBS can activate multiple cellular and molecular pathways, including BDNF-TrkB signaling, regulation of glutamatergic neurotransmission, and calcium-dependent plasticity mechanisms. These pathways collectively promote synaptic remodeling and functional recovery (14, 15). In SPG11, CST degeneration reduces cortical excitability and descending motor drive, necessitating interventions that potentiate–rather than suppress–cortical output (16). High-frequency repetitive transcranial magnetic stimulation (rTMS) is a promising therapeutic approach for patients with SPG11 because it can regulate cortical excitability and facilitating motor recovery (17). As an optimized rTMS paradigm, intermittent theta-burst stimulation (iTBS) induces more durable long-term potentiation-like effects over a shorter treatment duration (18). Conversely, continuous theta-burst stimulation would be mechanistically incongruent with the therapeutic goal in CST degeneration, as it suppresses cortical excitability via long-term depression-like mechanisms (19). Functional electrical stimulation (FES) activates peripheral nerves and muscles via controlled currents to evoke coordinated contractions, reinforcing spinal sensorimotor integration and remodeling maladaptive neuromuscular synergy patterns through proprioceptive feedback (20). When combined with central neuromodulation, FES forms a bidirectional intervention framework that complements “top-down” corticospinal modulation with “bottom-up” peripheral sensory input, potentially amplifying neuroplastic effects (21).

The combined use of rTMS and FES has been reported to facilitate hand function recovery in patients with stroke (22). The combined use of iTBS and FES has also been reported to improve gait function and activities of daily living of patients with spinal cord injury (23). However, to date, no study has investigated the combined use of iTBS and FES as adjuncts to ITB in patients with SPG11. In this report, we describe the case of a patient with SPG11 who received a multimodal regimen comprising ITB. iTBS, FES, and conventional rehabilitation. We evaluated changes in spasticity, muscle strength, balance, and psychological status and explored the potential clinical utility of this combined strategy for HSP rehabilitation.

## 2 Case description

### 2.1 Patient information

A 23-year-old female patient was admitted to Shanghai Eighth People's Hospital 2 weeks post-ITB surgery, presenting with a decade-long history of progressive lower-limb weakness and gait instability. Since the age of 13 years, she has displayed an abnormal gait, resulting in accelerated wear of footwear on the medial forefoot. By the age of 15, she had experienced difficulties descending stairs and required assistance for ambulation. After walking 500 m, she required rest. At 21 years of age, she began using a walker and exhibited a notably slow and laborious walking pattern, needing to pause after traversing approximately 20 m. The patient struggled academically throughout her childhood, achieving average to below-average grades in primary school (averaging approximately 70 points) and significantly lower scores in junior high school, with an average of 30 points.

Genetic testing performed at a Beijing hospital 1 year after symptom onset revealed compound heterozygous mutations in the *SPG11* gene, confirming the diagnosis of HSP type 11. Initially, the patient noted an improvement in gait heaviness with baclofen treatment. However, the efficacy of the drug diminished over time, with a maximum dosage of 15 mg administered thrice daily. Ambulation progressively deteriorated, and cold temperatures frequently triggered severe lower-limb stiffness, rendering the patient unable to walk. The patient underwent successful ITB pump implantation at Shanghai Sixth People's Hospital and was transferred to our department for postoperative rehabilitation 2 weeks later.

This case report was approved by the Ethics Committee of Shanghai Eighth People's Hospital and was conducted in accordance with the Declaration of Helsinki. Written informed consent was obtained from the patient for the publication of this case report.

Upon admission to our medical facility, she was unable to stand or walk unassisted, significantly impeding her ability to perform daily activities effectively. Cognitive assessment revealed a Mini-Mental State Examination score of 19/30 and a Montreal Cognitive Assessment score of 10/30, indicating moderate cognitive impairment. The patient was fully conscious but presented with slow, dysarthric speech and brief responses. Although her orientation and comprehension were not entirely compromised, her ability to perform calculations was notably diminished. Neurological examination revealed bilateral hyperreflexia of the knee and ankle reflexes, bilaterally positive Babinski signs, and patellar and ankle clonus. Furthermore, a reduction in muscle strength was noted in both lower extremities along with a significant increase in muscle tone. The patient also exhibited mild anxiety and depression.

Magnetic resonance imaging (MRI) of the brain revealed a marked reduction in corpus callosum thickness, particularly in the genu and splenium regions. Furthermore, increased signal intensity was observed in the periventricular white matter, which is closely linked to SPG11 (16). These observations are depicted on an axial fluid-attenuated inversion recovery (FLAIR) image (Figure 1).

**Figure 1:**
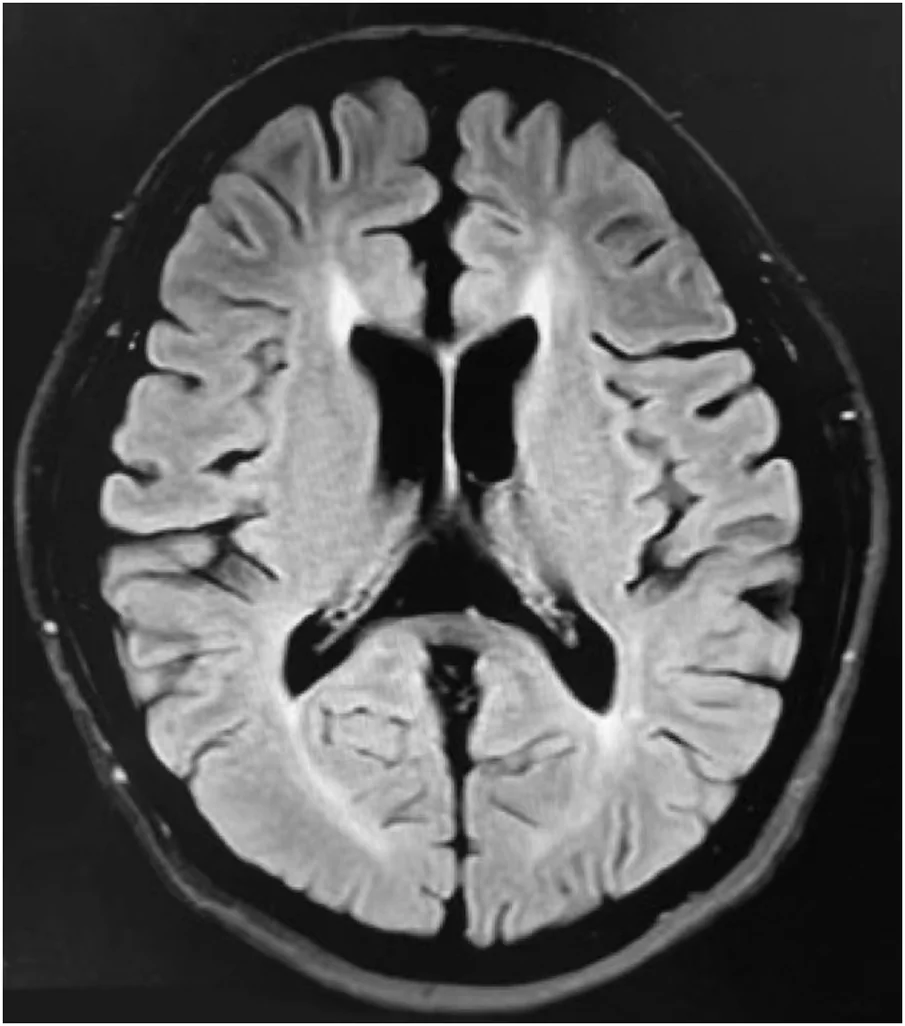
Magnetic resonance imaging of the brain. The image shows thinning of the corpus callosum and increased signal intensity in the periventricular white matter, findings consistent with HSP. The axial fluid-attenuated inversion recovery (FLAIR) image demonstrates the characteristic “ears of the lynx” sign, indicated by the white arrow.

### 2.2 Treatment method

A multidisciplinary team consisting of neurologists, neurosurgeons, and rehabilitation physicians developed a 3-month comprehensive rehabilitation plan, including dynamic ITB dose titration, neuromodulation therapy, FES therapy, and conventional rehabilitation. The intervention sequence was as follows: the patient underwent iTBS first, followed by FES, and finally conventional rehabilitation. No adverse events were observed during the study period. The patient tolerated the iTBS and FES interventions well, with no adverse events reported.

#### 2.2.1 ITB dosage titration

We aimed to balance spasticity control and functional performance during ITB dose adjustment. When the initial dose of ITB was 40 µg/d, the muscle tone in both lower extremities was excessively high. During rehabilitation, we gradually increased the baclofen pump dose to 61.18 µg/d. This change resulted in decreased muscle tone in the lower extremities, increased muscle strength, and improved balance. However, when we further increased the dose to 70.36 µg/d, the muscle tone in the lower extremities became too low, leading to decreased muscle strength and impaired balance. Consequently, we gradually reduced the baclofen dose to 62.82 µg/d, which led to a rebound increase in muscle tone. After comprehensive rehabilitation training, the patient was able to walk indoors using a walker. Before each dose escalation, potential noxious or irritative triggers of increased spasticity (e.g., urinary tract infection, constipation, or pressure ulcers) were excluded.

#### 2.2.2 Intermittent theta-burst stimulation

The intermittent theta-burst stimulation (iTBS) was delivered via a figure-eight coil connected to a magnetic stimulator (Yiruide, Wuhan, China). The iTBS protocol used 50 Hz intra-burst pulses repeated at an inter-burst frequency of 5 Hz. Each stimulation train lasted 2 s, and 600 pulses were delivered to each hemisphere per session, with the two hemispheres stimulated sequentially, corresponding to a total treatment duration of 3 min 20 s per hemisphere (23). The resting motor threshold (RMT) was measured before the first iTBS session. The stimulation intensity was set at 67% of RMT based on the patient's tolerance, which corresponded to 28% of the maximum stimulator output (MSO) in this patient. The RMT was defined as the minimal stimulation intensity capable of evoking a motor-evoked potential ≥50 *μ*V in the relaxed first dorsal interosseous (FDI) muscle across at least 5 out of 10 trials (24). The coil was positioned over the bilateral leg motor cortex (M1-LEG), 1–2 cm anterior to the vertex. Treatment was administered once daily, 5 days per week, for three consecutive months. No adverse events were reported during iTBS. During each iTBS session, adverse events were monitored using a structured questionnaire covering known TMS-related symptoms, including headache, scalp discomfort, dizziness, nausea, and abnormal sensations. The patient was also encouraged to report any unexpected symptoms to the research team at any time. No adverse events were reported during iTBS.

#### 2.2.3 Functional electrical stimulation

FES was delivered via a biofeedback therapeutic device (SW3000, Guangzhou Shirui Medical Technology Co., Ltd., Guangzhou, China). The treatment parameters were slightly adjusted based on previous studies (25). Surface electrodes were placed bilaterally over the quadriceps, tibialis anterior, and gluteus maximus. A biphasic square waveform was delivered at a frequency of 30–50 Hz, with the intensity adjusted between 10 and 100 mA to induce visible muscle contractions without patient discomfort. Each session lasted 20 min and was administered once daily. Stimulation parameters were periodically adjusted to accommodate neuromuscular adaptation and maintain therapeutic efficacy.

Following FES, the patient completed conventional rehabilitation therapy, including physical therapy, speech therapy, and ambulation training using a walker.

### 2.3 Clinical assessment and results

The patient underwent rehabilitation assessments on the day of admission and at 3 months post-intervention. All outcome assessments were conducted by trained physiotherapists who were blinded to the intervention to reduce potential measure bias. The primary outcome measures were muscle tone, measured using the Modified Ashworth Scale (MAS), and muscle strength, measured using manual muscle testing (MMT). Secondary outcome measures included the Berg Balance Scale (BBS), postural control assessed with BioFlex-FP100 (Guangzhou Longest Medical Technology Co., Ltd., Guangzhou, China), and anxiety and depression evaluations. The MMT and MAS assessments were used to evaluate three specific joints in both lower limbs: the hip (including flexion and extension), the knee (encompassing flexion and extension), and the ankle (focusing on dorsiflexion and plantar flexion). The MAS uses a six-grade scale (0, 1, 1+, 2, 3, and 4), with higher grades indicating greater spasticity (26). The MMT uses a six-point scale (0–5), with higher grades indicating greater muscle strength (27). The BBS consists of 14 tasks assessing static and dynamic balance ability. Scores range from 0 to 56, with higher scores indicating better balance (28). BioFlex-FP100 is a posture control evaluation system that uses biomechanical feedback to quantify balance and stability. In this study, we report the trajectory length of the center of pressure (COP) during quiet standing, expressed in centimeters (cm), as a measure of overall postural stability. A shorter COP trajectory length indicates better postural stability. The Hamilton Anxiety Scale (HAMA) comprises 14 items that assess both mental and physical symptoms of anxiety (28). Scores range from 0 to 56, with higher scores indicating greater anxiety severity. In contrast, the Hamilton Depression Scale (HAMD) measures depressive symptom intensity (29), with higher scores indicating greater depressive symptom severity. The HAMA and HAMD scores were evaluated at both admission and discharge.

As shown in Table 1, compared with baseline values at an ITB dose of 40 μg/d, MAS scores decreased, and MMT scores increased, and standing balance improved at the 3-month post-treatment assessment at an ITB dose of 62.82 μg/d. In addition, HAMA and HAMD scores decreased (Table 2).

**Table 1:** Changes in MAS, MMT, BBS and COP measurements before and after treatment.

Item		Before treatment	After treatment
ITB dose		40 μg/d	62.82 μg/d
MAS-R	Hip flexors	2	1
	Hip extensors	3	1
	Knee flexors	2	0
	Knee extensors	2	1
	Ankle dorsiflexors	1	0
	Ankle plantarflexors	2	1
	Total	12	4
MAS-L	Hip flexors	2	1
	Hip extensors	3	1
	Knee flexors	2	0
	Knee extensors	2	1
	Ankle dorsiflexors	1	0
	Ankle plantarflexors	2	1
	Total	12	4
MMT-R	Hip flexors	1	3
	Hip extensors	1	4
	Knee flexors	2	4
	Knee extensors	1	4
	Ankle dorsiflexors	1	3
	Ankle plantar flexors	2	4
	Total	8	22
MMT-L	Hip flexors	1	3
	Hip extensors	2	4
	Knee flexors	2	4
	Knee extensors	1	4
	Ankle dorsiflexors	1	3
	Ankle plantar flexors	2	4
	Total	9	22
BBS		19	26
COP trajectory length (cm)		25.3	22.9

**Table 2:** Results of the HAMA and HAMD measurements.

Item	pre-treatment	post-treatment 3 months
HAMA	10	5
HAMD	10	6

HAMA, Hamilton Anxiety Scale; HAMD, Hamilton Depression Scale.

## 3 Discussion

To our knowledge, this is the first report describing the combined use of invasive ITB, iTBS, and FES in a patient with genetically confirmed SPG11. Our findings demonstrate that this multimodal regimen led to clinically meaningful improvements across multiple domains. Spasticity markedly decreased (total MAS: 12 → 4), muscle strength increased substantially (total MMT: 8/9 → 22), balance improved (BBS: 19 → 26), and postural stability was enhanced (COP trajectory length: 25.3 → 22.9 cm). Moreover, the patient's anxiety and depression scores both showed notable reductions. These multi-domain improvements suggest that the combined ITB–iTBS–FES regimen may overcome the functional ceiling traditionally observed with ITB monotherapy in HSP, providing a novel rehabilitation strategy for SPG11.

A notable clinical observation in this case was an apparent dissociation between ITB dose and functional outcomes. At the initial dose of 40 μg/d, severe spasticity (MAS total 12 per side) severely restricted voluntary movement and balance. Increasing the dose to 70.36 μg/d excessively reduced muscle tone, resulting in paradoxical functional decline. Specifically, muscle strength decreased and BBS scores dropped, suggesting that the near-total abolition of spasticity may prove counterproductive by removing compensatory postural stability needed for motor re-education in patients with underlying muscle weakness. The best observed balance between spasticity control and functional performance occurred at 62.82 μg/d, when spasticity control was sufficiently controlled to allow movement while residual tone was preserved to support weight-bearing and dynamic balance. Although these findings are limited to a single case and cannot establish a definitive dose–response relationship, they align with previous studies emphasizing that ITB dosage should be titrated based on functional outcomes rather than spasticity scores alone (30). This underscores the importance of close collaboration between neurosurgical and rehabilitation teams to dynamically adjust ITB parameters and align spasticity management with motor re-education goals.

Because ITB reduces spinal spasticity but does not address supraspinal dysfunction, adjunctive strategies that engage cortical and peripheral components of the motor network may be beneficial. The combination regimen utilized in this study operates on three interconnected levels: ITB optimizes spinal circuit excitability to create a permissive “neuroplastic window”; iTBS enhances top-down corticospinal drive through long-term potentiation-like effects on the primary motor cortex; and FES provides bottom-up proprioceptive feedback to reinforce spinal sensorimotor integration. This multimodal framework was intended to engage both spinal and supraspinal components relevant to motor recovery.

The choice of iTBS over cTBS was based on their distinct neurophysiological effects and the pathophysiology of SPG11. iTBS induces LTP-like facilitation of cortical excitability, whereas cTBS induces LTD-like suppression (18). Given that SPG11 is characterized by progressive CST degeneration, the therapeutic goal was to strengthen–rather than dampen–residual corticospinal transmission. Thus, iTBS is mechanistically aligned with this objective, whereas cTBS would be less aligned with the intended excitatory neuromodulatory effect. This rationale is consistent with previous reports that HF-rTMS has been reported to improved motor function in SPG11 (17) and that iTBS has been applied in conditions with corticospinal dysfunction (31). Accordingly, iTBS was selected to potentiate residual CST connections in conjunction with ITB and FES.

The concurrent use of iTBS and FES may have contributed to the observed functional improvements through synergistic neuroplastic mechanisms acting at multiple levels of the neuraxis. At the cortical level, iTBS modulates glutamatergic neurotransmission by enhancing NMDA receptor-dependent signaling pathways, particularly CaMKII phosphorylation and AMPA receptor trafficking (32). This may increase cortical excitability and strengthen corticospinal synapses, as evidenced by previous DTI studies demonstrating increased CST fractional anisotropy following excitatory rTMS (33, 34), and meta-analyses confirming that rTMS significantly increases motor-evoked potential amplitudes in patients with neurological disorders (35). Furthermore, NIBS has been shown to promote BDNF signaling, which serves as a central hub for synaptic plasticity and neuronal survival (36). Previous evidence has suggested that iTBS, when combined with antioxidant and anti-inflammatory strategies, may synergistically reduce oxidative stress and enhance neuroplasticity, providing a translational framework for multimodal neurorehabilitation (37). At the spinal level, FES synchronously activates *α*-motor neurons and Ia afferent fibers, delivering real-time proprioceptive feedback that reinforces sensorimotor integration (38). The timed arrival of FES-induced afferent volleys to the motor cortex may reinforce corticospinal synapses via spike-timing-dependent plasticity, wherein peripherally and cortically derived inputs converge temporally to facilitate synaptic strengthening (39, 40). This hierarchical neuromodulation may help explain the observed improvements in MMT and BBS scores, as residual corticospinal connectivity may have been potentiated while peripheral muscle recruitment was optimized.

The marked reductions in anxiety and depression scores observed in our patient occurred alongside the improvements in motor function and balance. Epidemiological data show that up to 60% of patients with HSP develop clinically significant mood disorders associated with chronic spasticity and a progressive loss of autonomy (41). Our multimodal intervention interrupted the spasticity-anxiety feedback loop by relieving persistent muscular discomfort and restoring self-efficacy through regaining ambulation capacity and reducing caregiver dependence. These motor-driven psychological gains further boost rehabilitation adherence, creating a virtuous cycle that amplifies overall therapeutic benefits.

Despite these encouraging observations, this study has some limitations. First, the single-patient design limits the generalizability of the findings. Second, the 3-month follow-up period was insufficient to evaluate the long-term sustainability of the treatment effects in this progressive disorder. Third, the outcome assessments–particularly MMT and MAS–are inherently subjective and may introduce measurement bias despite standardized training. Fourth, because ITB titration was performed concurrently with the iTBS-FES intervention, changes in baclofen dose may have influenced spinal and cortical plasticity and confounded attribution of the observed functional changes. Increasing the ITB dose to 70.36 μg/d produced excessive hypotonia and paradoxical functional decline, whereas optimal gains occurred only after the dose was reduced to 62.82 μg/d in conjunction with continued iTBS and FES. Although the apparent dissociation between ITB dose and functional outcomes suggests that ITB dose alone may not fully explain the functional trajectory, the respective contributions of ITB, iTBS, and FES cannot be separated in this single case. Nevertheless, to reduce this potential confounding, future studies should consider delivering the neuromodulation protocol after achieving a stable ITB maintenance dose. Fifth, the RMT was determined from the FDI muscle, whereas stimulation was delivered over the leg motor cortex. Because corticospinal excitability and motor thresholds differ substantially between hand and lower-limb muscles—with leg representations being deeper and more medial, and thus requiring higher intensities—an FDI-derived RMT may not accurately reflect the intensity required for M1-LEG. Future studies should determine RMT directly from lower-limb muscles such as the tibialis anterior. Future controlled trials with larger samples are needed to evaluate efficacy, feasibility, and safety; optimize treatment parameters; and identify predictive biomarkers of treatment response.

## 4 Conclusions

This case suggests that iTBS combined with FES may serve as a potential adjunct to ITB in patients with SPG11 and may be associated with improvements in spasticity, motor function, balance, and psychological symptoms. Dynamic interdisciplinary titration of the ITB dose may help balance spasticity control and motor learning. By combining cortical and peripheral neuromodulation approaches, this strategy warrants further investigation. Future studies should evaluate its efficacy, feasibility, and safety and optimize treatment before broader clinical application.

## Data Availability

The raw data supporting the conclusions of this article will be made available by the authors, without undue reservation.
